# Volume and outcome relation in German liver transplant centers: what lessons can be learned?

**DOI:** 10.1186/2047-1440-3-5

**Published:** 2014-02-10

**Authors:** Annemarie Nijboer, Frank Ulrich, Wolf O Bechstein, Andreas A Schnitzbauer

**Affiliations:** 1 Frankfurt University Hospital, Goethe-University Frankfurt/Main, Clinic for General- and Visceral Surgery, Theodor-Stern-Kai 7, Frankfurt/Main 60590, Germany

## Abstract

**Background:**

The volume and outcome relationship for transplant procedures has become one of the major topics during discussions about consequences following the organ transplantation scandal of wait-list manipulations in Germany during the past year. Proponents of reducing the number of centers argue in favor of increasing quality at highly specialized transplant centers while disregarding the wish of patients for regionally available medical service.

**Methods:**

The homepage of the German Organ Procurement Organization (DSO) was screened for the annual reports of transplant programs for the years 2007 to 2010. Results were extracted from these reports. Additionally, an analysis of volume per million people per number of transplant centers for each German federal state was made to give an overview of the density of transplant programs for the years 2009 to 2011.

**Results:**

In-house mortality (R^2^ = 0.005, *P* = 0.518), 3-year survival (R^2^ = 0.068, *P* = 0.085), and a ROC analysis for in-house mortality (AUC 0.55, CI: 0.41; 0.68, *P* = 0.53), did not show volume-outcome relation. Definition of a threshold for good centers was impossible. One-year survival indicated better outcome in high volume centers. R^2^ = 0.106, *P* = 0.009. Outcome data in Germany, as provided by Institute für angewandte Qualitätsförderung und Forschung im Gesundheitswesen (AQUA) or the DSO, are not risk adapted for the investigated time period. The factor of transplants per year per million people per transplant centers is 0.6 for Germany. Some Federal States (for example, Bavaria and Northrhine Westfalia) have an oversupply of transplant centers, which means that the average number transplanted per center and year is very low.

**Discussion and conclusion:**

We propose a risk-adapted prospective analysis of outcome and definition of a quality catalogue for liver transplant centers. Volume and outcome relation is not conclusive for liver transplantation in Germany. Data should be collected, for example, for a time period of 3 to 5 years, and decisions influencing the regulation of numbers of transplant centers should be based upon the findings, weighing federal state sovereignty and regional medical requirements against an optimal patient supply while respecting a plausible risk adaption for each center.

## Introduction

The German transplant scandal in 2012, which uncovered several centers with wait-list manipulations indicating higher than actual urgency in patients, unleashed a variety of controversies and debates on the current system of organ transplantation. Among very reasonable and politically important topics, like the discussion of the current organ allocation system, self-administration and transparency in maintenance of wait lists as established in Germany and represented by the German Medical Association and the call for more governmental control, there were also strong voices that demanded a reduction of the currently 24 centers performing liver transplantations in Germany. One of the leading arguments for reducing the number of centers was an increasing quality of liver transplantation by focusing on centralization on a small number of high-volume centers. Although, some evidence for volume-outcome relation has been shown for various surgical fields, including liver transplantation, it is quite difficult and challenging to define proper thresholds for the size of 'good’ transplant centers [1]. Moreover, the field has undergone a dynamic change and has various confounding and bias-factors that may draw a misleading picture [1,2]. Opponents of closing down small transplant centers argue with the right for a regional medical supply for all patients in times of extreme rationalization and compression of medical treatment in particular areas of Germany. Proposals of centers delivering good outcome range between 20 to 50 liver transplants per year. In order to lift this element of the current debate from a perceived level of evidence to a level with resiliently arguable data based on the best available information, we performed an analysis of in-house mortality, 1- and 3-year survival analysis from annual reports of the German Organ Procurement Organization (DSO) in relation to the volume of transplant centers in Germany for the years 2007 to 2010.

## Methods

### Data source and extraction of data

To receive data for each liver transplant center, the homepage of the Deutsche Stiftung für Organtransplantation (DSO) was screened for the annual reports of transplant programs for the years 2007 to 2010 [3]. From these reports the results for every individual liver transplant program in Germany were extracted as published in the report under point 1.5 in 'results of transplantation’. Data collected were: 1) patients reported and in-house mortality, 2) 1- and 3) 3-year mortality rates of the patients reported to the DSO in the particular years, and 4) total number of patients transplanted. In cases where five or fewer patients were reported to the DSO, the number of patients was set to five since an exact number of patients reported was not documented in the reports for data safety protection regulations.

Moreover, cumulatively presented data for the years 2009 to 2012 were collected from the annual reports for liver transplantation, which are published by the AQUA institute, the organization that is officially responsible for quality control in liver transplant programs. These data were available from the homepage (https://www.sqg.de/ergebnisse/leistungsbereiche/lebertransplantation.html) and were compared to the data from the DSO to determine consistency of the data extracted from the DSO homepage.

Additionally, an analysis of volume per million people per number of transplant centers for each individual German federal state was made to give an overview of the concentration and density of transplant programs in each particular state. Data of the number of liver transplantations from deceased donors for the years 2009 to 2011 were extracted from the DSO homepage (http://www.dso.de/medien-und-presse/pressebilder-und-grafiken.html). The number of people living in each federal state was extracted from the homepage of the Federal Office of Statistics in Germany.

### Analysis

In-house mortality and 1- and 3-year survival data were analyzed descriptively. Data are given as the mean with standard deviation and median with ranges along with 95% confidence intervals (95% CI). Linear regression analyses, analogous to the analysis of Edwards *et al*., were performed to identify a correlation of survival and size of the transplant centers [4]. R^2^ values (Pearson correlation) and levels of significance (*P* values) were depicted and interpreted. Nonlinear regression analysis was performed for in-house mortality with a polynomial cubic equation via dynamic fitting to exclude significance with another model. Additionally, for in-house mortality, a receiver operating characteristic (ROC) analysis was performed, assuming that an in-house mortality of less than 20% is acceptable as demanded in the annual quality definitions for liver transplantation of AQUA (Institut für angewandte Qualitätsförderung und Forschung im Gesundheitswesen, Göttingen, Germany). Data are given as area under the curve (AUC) with 95% CI and significance levels. The outcome for volume per million people per number of transplant centers in the individual federal states in Germany was given as a factor. Statistical analysis was performed with SPSS™ Version 20 (IBM, Armonk, New York, USA).

## Results

### Completeness and quality of data

For a total of 3,317 patients, in-house mortality rates were reported whereas a total of 4,316 patients were reported as transplanted in the investigated 3-year period. This resulted in a reporting rate of 76.9% of patients, reflecting the large variety of the quantity of reported data in the investigated time period between 2007 and 2010. Notably, quality control of liver transplant programs does not distinguish between transplantation from deceased donors, living-related liver transplantations, pediatric liver transplantation or split-liver transplantation, so far. Information on the number of re-transplantations was also not available.

### In-house mortality

**Figure 1:**
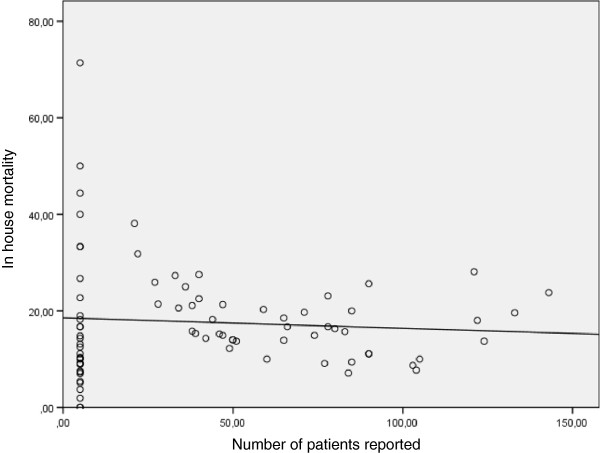
**In-house mortality for German liver transplant centers per center per year including linear regression analysis.** R^2^ = 0.005, *P* = 0.518.

**Figure 2:**
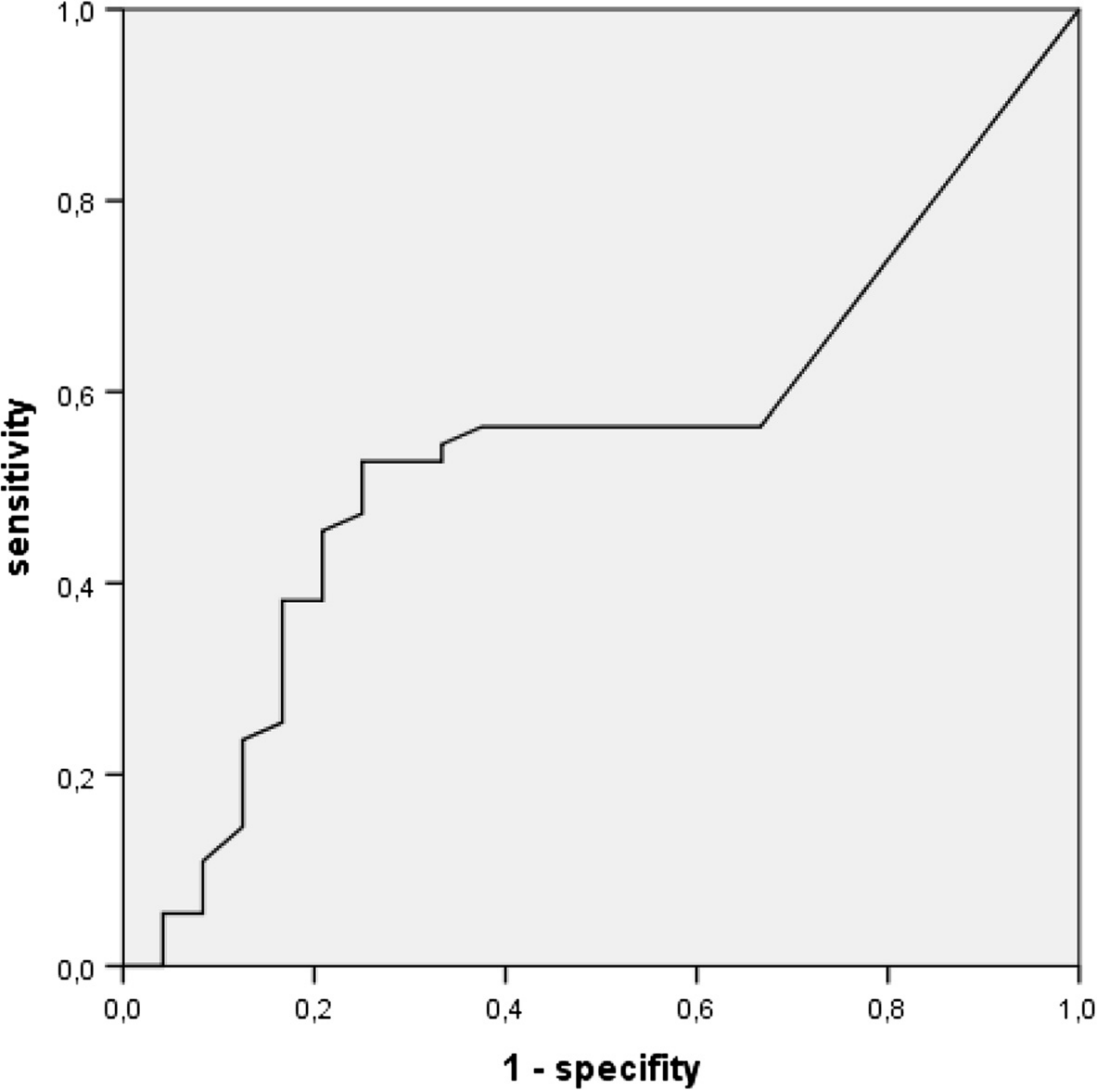
**Receiver operating characteristics (ROC)-analysis for in-house mortality without delivering a threshold, indicating a volume outcome relation.** (AUC 0.55, CI: 0.41; 0.68, *P* = 0.53).

A total of 79 (82%) of 96 datasets (1 report per center per year from 24 centers) reporting in-house mortality were available for the years 2007 to 2010. A total of 24 transplant centers reported their results to the DSO. The mean in-house mortality was 17.6 ± 11.3% (range: 0 to 71.4%). The German institute for quality control AQUA defined an in-house mortality of ≤20% as acceptable. In-house mortality >20 percent precipitated queries for explanations of mortality from the particular center for every individual case to a monitoring board at AQUA. Figure 1 shows an equally distributed cloud diagram around the 20% in-house mortality line for small centers (but reporting more than five liver transplants per year) as well as for larger centers. This is supported by the ROC-analysis (AUC 0.55, CI 0.41; 0.68, *P* = 0.53) displayed in Figure 2, delivering no clinically significant cut-off value for the volume of a transplant center that impacts transplant outcome positively or negatively. Notably, there are a large number of centers that reported five patients or fewer, although officially performing much higher numbers of liver transplants. The number of centers performing fewer than 20 liver transplants per year was stable over the years, ranging between 4 and 8 (16.7 to 33.3%) centers.

### One- and three-year patient survival

**Figure 3:**
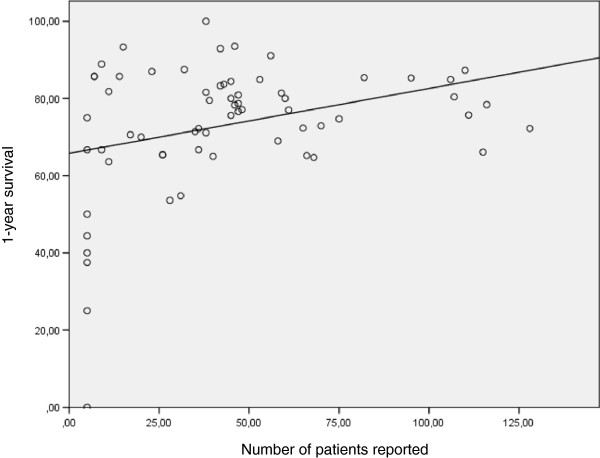
**One-year survival for German liver transplant centers per center per year including linear regression, indicating better outcome in high volume centers albeit reduced quality of data.** R^2^ = 0.106, *P* = 0.009.

**Figure 4:**
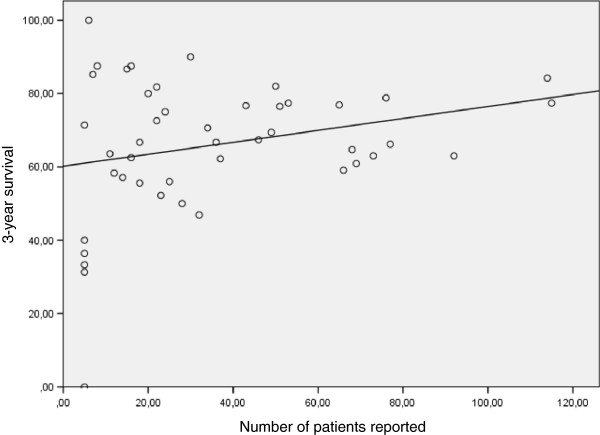
**Three-year survival for German liver transplant centers per center per year including linear regression, indicating no relation between volume and outcome albeit reduced quality of data.** R^2^ = 0.068, *P* = 0.085.

For 1-year survival data a total of 64 (67%) of 96 datasets were reported to the DSO. One-year overall mean survival was 73.2 ± 16.8% (range: 0 to 100%). Figure 3 shows a cloud diagram with an equal distribution for transplant centers reporting more than five transplants per year. Three-year survival was reported with a mean of 66.0 ± 18.4% (range: 0 to 100%) reflecting a trend for better outcome in sites transplanting more than 30 livers per year (Figure 4). However, this finding is based on 45 (47%) of 96 datasets received during the investigated period.

### Linear regression analysis comparing outcome and volume

Regression analysis for in-house mortality (r^2^ = 0.007, *P* = 0.52) and 3-year overall survival (r^2^ = 0.05, *P* = 0.09) delivered no significant correlation between center volume and outcome. However for 1-year-overall survival, a better outcome was significantly correlated with larger center size (r^2^ = 0.09, *P* = 0.009). Nonlinear regression, as mentioned in the methods section, did not reveal a significant relationship between parameters for in-house mortality (*P* = 0,257).

### Consistency check of results comparing DSO data with AQUA institute data

A critical review of the findings of our analysis suggests that data quality may cause a significant bias. Therefore, overall quality data from the AQUA institute were excerpted from the annual reports as a consistency check. The completeness of reported datasets to the AQUA institute for the years 2009 to 2012 was between 96 and 103% for all liver transplant programs in Germany as outlined in their reports. The median in-house mortality for centers performing fewer than 20 liver transplants per year was similar to that for centers with more than 20 transplants per year, reaching less than 20% in-house mortality. Similar data were published for 1-year survival of patients who did not experience in-house mortality. In both groups, regardless of the number of transplants performed, the average 1-year survival rate censored for in-house mortality was between 90 and 93% for the years 2009 to 2012.

### Transplant volume per million people per transplant center

There were 3,413 liver transplants from deceased donors in Germany between 2009 and 2011, which is an average of 1,137 transplants per year performed at 24 transplant centers in Germany, representing 82 million people. Descriptively calculated, the annual number of transplants per million people per transplant centers is 0.6 in Germany. However, when calculated as the number of transplants per million people per transplant centers in each individual state, results become clearer and depict a representative density of regional medical supply. Considering these calculations, there are regions with fewer than 5 transplants per million people per transplant centers (Bavaria, Northrhine-Westfalia, Mecklenburg-Western Pomerania) and other regions between 5 and 15 transplants per million people per transplant centers (Baden-Wurtemberg, Hesse, Lower Saxony, Rhineland-Palatinate, Saxony-Anhalt, Schleswig Holstein/Hamburg) and those with more than 15 transplants per million people per transplant centers (Berlin/Brandenburg, Saarland, Saxony, Thuringia) (Table 1). Excluded from our consideration was a certain patient traffic from one federal state to the other, especially in adjacent regions or over-regionally active sites, who recruit their patients from all over Germany. Nonetheless, when comparing the numbers with data from the United Kingdom (UK) (as outlined in the discussion), data appear to be consistent, comparable and conclusive.

**Table 1:** Overview of liver transplant centers per federal states and the number of transplants per million

**Federal State**	**Population in Million**	**LTx programs**	**LTx (Living donor liver transplants excluded)**			**LTx/Million people/number of centers**
			**2011**	**2010**	**2009**	
**Bavaria**	12.6	5 (4 in 2009) (Munich (2), Regensburg, Erlangen, Würzburg)	156	166	152	**2.7**
**Baden-Wuertemberg**	10.8	2 (Heidelberg, Tübingen)	134	135	145	**6.5**
**Berlin**^**e**^**/Brandenburg**^**f**^	6.0	1 (Charité Berlin)	93	102	116	**17.3**
**Hesse**	6.1	1 (Frankfurt/Main)	29	46	37	**6.1**
**Mecklenburg-Western Pomerania**	1.6	1 (Rostock)	5	5	0	**2.1**
**Lower Saxony**^**b**^**/Bremen**^**a**^	7.9	2 (Hannover, Göttingen)	112	145	139	**8.4**
**Northrhine Westfalia**	17.8	5 (4 in 2009) (Essen, Aachen, Münster, Cologne, Bonn)	260	230	214	**2.8**
**Rhineland-Palatinate**	4.0	1 (Mainz)	43	43	42	**10.8**
**Saarland**	1.0	1 (Homburg)	15	31	9	**18.0**
**Saxony**	4.1	1 (Leipzig)	97	85	79	**21.2**
**Saxony-Anhalt**	2.3	1 (Magdeburg)	20	19	7	**6.5**
**Schleswig-Holstein**^**d**^**/Hamburg**^**c**^	4.6	2 (Hamburg, Kiel)	118	107	125	**12.7**
**Thuringia**	2.2	1 (Jena)	49	56	47	**23.2**

People per number of transplant centers for each individual state/region in Germany. Bremen^a^, having no own medical faculty was added to Lower Saxony^b^. Hamburg^c^ and Schleswig Holstein^d^ as well as Berlin^e^ and Brandenburg^f^ were combined to one major region each.

## Discussion

The relation of volume and outcome for German liver transplant centers cannot be conclusively and satisfactorily determined. Data provided by the DSO, as well as by the AQUA institute, are not strikingly significant for all years analyzed. There are several reasons for this conclusion. First, the quality of reported data is moderate with completeness of datasets reaching only 82% for in-house mortality and less for 1- (67%) and 3- (47%) years overall survival for the years 2007 and 2010. To receive a clear picture a 100% quality assurance is necessary. Otto *et al*. indicated this discrepancy and problem earlier, in 2011, when they drew a worst-case and best-case scenario for 1-year survival data after liver transplantation. For the year 2009 a 1-year survival of 76.1% was reported by the AQUA institute. However, the status of 165 out of 964 patients was unknown, resulting in a best-case scenario (all patients with unknown status alive) of 80.2% and a worst-case scenario (all patients with unknown status dead) of 63.1% [5]. The current situation in Germany is that the AQUA institute demands 100% data reports and paints a worst-case scenario in case of missing data, indicating lower quality for noncompliant centers. However, data from the AQUA institute, as outlined in the results section for the years 2009 to 2012, also did not reveal noticeably better results - except for 1-year survival in 2012 - for larger centers with a data quality ranging between 96 and 100% for the investigated years. Therefore, our findings are consistent albeit data quality for individual centers from the DSO-homepage is moderate.

Second, results for pediatric and adult liver transplantation are reported as one collective, although results vary significantly, reaching 1-year survival rates of 90 to 95% for pediatric liver transplant recipients [6]. This leads to a possible bias in the interpretation of better results from centers that perform pediatric and adult liver transplantation. For Germany, results of adult transplantation are inferior to data from other countries: a multicenter analysis by Weissmuller *et al*. showed a dramatically worse outcome for liver transplant recipients in the model for end-stage liver disease (MELD) era with declining results in the MELD >30 group [7]. More than 40% of all patients are transplanted in a status of high urgency or with a MELD >30 in Germany [8,9]. The rules apply uniformly for all centers in the same way. Moreover, results are not risk adapted for indications. There may be several centers that perform more transplantations with a higher risk and known inferior outcome after liver transplantation, such as patients with hepatitis C virus (HCV) cirrhosis. Other centers may perform more split liver procedures or accept more extended criteria donors and therefore utilize 'unwanted organs’, which should also be acknowledged in a risk-adapted outcome report. In our opinion, and in accordance with UNOS-practice, results for pediatric and adult liver transplant programs should be reported separately from each other and should additionally be presented in a raw and risk adapted fashion. Notably, in this analysis, split procedures and living related transplantations (10% per year) were constant and not considered separately in this analysis.

Our analysis did not deliver a statistically meaningful cut-off value for the size of a liver transplant program that is directly associated with beneficial outcome. However, regression analysis indicated a volume and outcome relation for 1-year overall survival when transplant centers performed a higher number of liver transplants. This was confirmed by the AQUA institute analysis providing good to very good data quality and especially showing a more beneficial outcome for larger centers at 1 year after transplantation in 2012. General recommendations for the minimum size of liver transplant centers for providing satisfying outcome vary between 20 and 50 liver transplants per year [4,10,11]. However, there is a broad consensus that scientific reports simply correlating volume and outcome (= survival) are very vulnerable to confounding factors and that variations in results become large and very difficult to interpret [2].

A more specific look into existing literature assists in overcoming the pure one-dimensional approach to volume and outcome relation. Northup *et al*. analyzed 9,909 adult liver transplant recipients who were transplanted in the United States (US) after introducing MELD-allocation. They concluded that there is no longer a volume outcome relation for US liver transplant centers. Raw mortality was lower at large centers. But when factors like disease severity and donor and recipient factors were introduced (= risk adaption), the significance was no longer present between low and high volume liver transplant centers [12]. This means that by selection of recipients as well as donors, results - even at smaller sites - may be beneficial for patients requiring a life saving liver transplantation. *Vice versa*, high volume centers that accept more extended donor criteria organs can obtain satisfying results in their patients and thus utilize the whole available donor pool [13]. Especially for areas in which high-end medical supply is of reduced local availability compared to metropolitan areas, this may be a Solomonic way of providing optimal medical care for the majority of people in forms of smaller transplant centers, which have proven that they can also be successful [11].

To explain this in a more detailed fashion, we analyzed the number of transplants per million people per number of liver transplant centers. In Germany this factor is 0.6, which is less than half compared to the UK with 1.5. In the United Kingdom, numbers of liver transplant centers are limited to currently 7 sites performing 642 liver transplants for 63 million people in 2011 [14]. The factors for each individual region range between 3.6 and 12.8 transplants per million people per number of centers in a region, with only one single region performing less than 5 transplants per year per million people. In Germany there are regions that have five (Bavaria and Northrhine Westfalia) liver transplant centers with factors of 2.7 and 2.1 in the regional supply. Other centers reach factors of 6.5 to 23.2. However, reasons for low factors may be that there are too many transplant centers for too few people or too few liver transplants by single centers in a region with many people. The current situation, however, seems to uncover a heterogeneity in the regional plans of supply with liver transplantations throughout Germany, perhaps owing to the still prestigious and also financially relevant factor for hospitals to run even small transplant programs in highly competitive federal states. Single federal states (for example, Baden-Wurtemberg and Hesse), in whose hands the hospital requirement plan generally is placed by law, decided to limit the number of their liver transplant centers years ago. In other regions, the existence of a single University Hospital - with general ability of providing the required infrastructure to run a liver transplant program - do not face the problem of competing institutions within a Federal State. Other federal states, such as Bavaria or Northrhine-Westfalia, have not yet regulated the number of transplant centers.

## Conclusion

There has to be a consistent and persisting definition of quality for German liver transplant programs that will allow actions on liver transplant centers in case of failure. We propose the definition of a quality catalogue that includes accuracy of reporting to AQUA, success rate represented by 1-year patient and graft survival data [15], incidence of re-transplantations and audit results. Certification of centers with an evaluated minimum standard infrastructure, structured education of transplant specialists and reconsideration of budgeting of transplant medicine are further important steps that need to be accomplished. A data basis for a time period of, for example, 3 to 5 years, should be collected prospectively and decisions based upon the findings. Risk-adaption of confounding factors should be performed to present a clearer picture of outcome quality. By this strategy, of balancing urgency and success as defined in the German transplant legislation, the average lab MELD score at the time point of transplantation may be reduced because outcome will be a major focus of centers in indicating transplantations [8]. We further propose to report results from pediatric liver transplant programs separately from adult liver transplantation. Politicians and medical representatives are encouraged to explore an optimal balance between regional supply of medically underprivileged regions and a reasonable number of transplant centers delivering the best quality medical care for the population. This may also include highly specialized centers that for example focus on complex treatment of oncologic patients in which a treatment option is transplantation (model of the Italian Cancer Institute of Milan). Only by improving outcome through amending the current system, optimizing patient care and allocation practice, will society be willing to support transplant medicine through donations of organs in case of brain death, as the most critical corner point in this system.

## Abbreviations

AQUA: Institute für angewandte Qualitätsförderung und Forschung im Gesundheitswesen; AUC: area under the curve; CI: confidence interval; DSO: German organ procurement organization; HCV: hepatitis C virus; MELD: model for end-stage liver disease; ROC: receiver operating characteristics; UK: United Kingdom; US: United States.

## Competing interests

The authors declare that they do not have any competing interests.

## Authors’ contributions

AN collected data and wrote parts of the manuscript, performed literature research. FU generated the idea of the project and gave significant intellectual input in revising the manuscript. WB generated the idea of the project, performed literature research and gave significant intellectual input in revising the manuscript. AS generated the idea of the project, collected, analyzed and interpreted data and wrote parts of the manuscript. All authors read and approved the final manuscript.
